# Quality of Life of Polish Patients with Lymphoma Treated Systemically

**DOI:** 10.3390/nursrep13040119

**Published:** 2023-10-09

**Authors:** Małgorzata Pasek, Janina Biel, Anna Goździalska, Małgorzata Jochymek

**Affiliations:** 1 Department of Nursing, Faculty of Health, University of Applied Sciences, 33-100 Tarnów, Poland; malgorzata_pasek@wp.pl; 2 Faculty of Health and Medical Studies, A. F. Modrzewski Krakow University, 30-705 Krakow, Poland; janina.biel@rydygierkrakow2.pl (J.B.); mjochymek@afm.edu.pl (M.J.)

**Keywords:** health-related quality of life, chemotherapy, Hodgkin’s lymphoma

## Abstract

Research on the quality of life has become of great importance. It is used by clinical researchers to compare the impact of treatment regimens on patients. The choice of treatment method may significantly depend on the patient’s opinion. A cross-sectional study was conducted using the method of a diagnostic questionnaire survey. The research tools were the authors’ questionnaire and the standardized WHOQOL-BREF. The study was conducted among patients with lymphoma, treated systemically. More than half of the surveyed patients assessed their overall quality of life as good (50%) and very good (6%), while the expressed satisfaction with health most often ranged from neutral (38%—neither good nor bad) to dissatisfactory (30%) and very dissatisfactory (6%). As regards the detailed domains, the area of physical functioning was rated the lowest, while for the remaining domains—psychological, social, environmental—values above average (60.38–64.30) were observed. Social support, particularly from the immediate family, resulted in a higher assessment of the quality of life. The occurrence of side effects related to anticancer treatment and the disease had a statistically significant impact on the decrease in the quality of life, particularly in the physical domain.

## 1. Introduction

Lymphoid cancers account for about 5% of all neoplasms [1]. It is estimated that 62.4% of cases are non-Hodgkin’s lymphomas (NHL) and about 8.2% are Hodgkin’s lymphomas (HL) [2]. As a group of diseases, they are in sixth place in terms of incidence in women and seventh in men. They occur in all age groups, but most often in the seventh and successive decades of life [1,2].

Regardless of the etiological factors, the pathogenesis of the disease involves a malignant transformation of lymphocytes and genetic instability. Chromosomal translocations are the most common genetic aberrations leading to the development of lymphomas [3]. The most common translocations occur on chromosomes 14 and 18. In addition to these mechanisms, there are also mutations where structural aberrations lead to abnormalities in the maturation and differentiation of lymphocytes or disorders in the cell cycle [2,3].

The prognosis for survival for B-cell lymphomas of high histological malignancy is about 90%, and for T-cell lymphomas about 50% [4]. Accurate assessment of the disease stage in patients with HL is of key importance for selecting the appropriate therapy [4,5].

Therapeutic management of lymphatic system diseases is carried out in oncohematology centers with specialized personnel and the possibility for rapid diagnosis [5]. The classification of lymphomas indicates different types of morphological origin and thus a wide spectrum of therapeutic activity [2]. In indolent lymphoma, the progression of symptoms is slow. Patients survive without treatment for many years. However, in aggressive lymphoma patients survive without treatment only up to several months. The treatment of lymphomas is most often selected based on stage, prognostic factors, and coexisting diagnoses [6,7,8,9,10]. Chemotherapy causes damage to normal cells, which is associated with the occurrence of many side effects. There are different goals in the treatment of lymphoma; in aggressive diseases the intention is to cure, but in indolent diseases, the goal is to obtain an asymptomatic patient. An equally important goal in the treatment of lymphomas is to maintain the patients’ quality of life at a good level, despite the burdensome treatment and side effects [11]. Systemic treatment is defined as therapy that the patient’s entire body is subjected to. Chemotherapy, immunotherapy, and hormone therapy all apply [11,12].

The literature describes studies on the quality of life of convalescents after treatment [12,13], showing that the suffering associated with physical symptoms, anxiety, depression, poor adaptation, and receiving chemotherapy disturbed their quality of life. In this aspect, it seems advisable to cover the entire path, from diagnosis to survival or recurrence/metastases, with quality-of-life research, the results of which can be used for direct patient care or may have a prognostic value in planning subsequent stages of recovery.

In the patient’s treatment process, the informal caregiver is crucial for ensuring sustainable oncological care; he or she often manages the patient’s care at home and in the hospital. His presence and the support have a direct impact on the patient’s assessment of the quality of life.

The concept of health-related quality of life (HRQL) has been present in medicine since the 1970s. Schipper defined it as the patient’s perception of the impact of the disease and its treatment on the functioning and general sense of life satisfaction [14]. The definitions of HRQL proposed by many authors [15,16] have a common feature, namely, the assessment is subjective and made only by the patient. Only standardized research tools should be used to measure the quality of life [17,18,19].

Research on the quality of life has now become of great importance. It is used by clinical researchers to compare the impact of treatment regimens on patients. The choice of treatment method may depend significantly on the patient’s opinion. It is risky to conclude from theoretical studies, even if they are of great research and clinical importance, if the occurrence of the side effects experienced by the patient in all aspects of life significantly exceeds the therapeutic effects.

Our study aimed to assess the quality of life of patients with lymphoma treated systemically in a hospital. Obtaining patients’ opinions regarding their views on the therapy process can become a valuable source of information for their direct and indirect caregivers and families or people who are significant to the patients.

To achieve the assumed research goal, we set out the following research problems:

(1) How do the surveyed patients assess their quality of life and health as well as their physical health, psychological condition, social relations, and the environment?
(2) How do demographic variables affect the respondents’ assessment of their quality of life?
(3) How does the support received affect the respondents’ assessment of their quality of life?
(4) How do the side effects of systemic treatment and comorbidities affect patients’ assessment of their quality of life?

## 2. Materials and Methods

### 2.1. Research Methods

A cross-sectional study was conducted using the method of a diagnostic questionnaire survey on a non-probabilistic convenience sample of 82 patients diagnosed with lymphoma.

The research tool was a questionnaire, which consisted of the authors’ part for the sociodemographic and clinical assessment of respondents, and the standardized World Health Organization Quality of Life Test-Bref (WHOQOL-BREF) for assessing the quality of life, consisting of 26 questions. The quality of life, in line with the authors’ assumptions, is assessed in six dimensions. The first two items concern the patient’s perception of their quality of life and general health, and the remaining four cover their physical health, psychological condition, social relations, and the environment. The assessment of physical health covers the following: pain and discomfort, drug and treatment dependence, energy and fatigue, mobility, rest and sleep, everyday activities, and the ability to work. The psychological domain includes positive feelings, spirituality, religion, personal faith, thinking, learning, concentration, appearance, self-esteem, and negative feelings. In the social domain, the following aspects are taken into account: personal relationships, sexual activity, and social support. The environmental domain concerns the following elements: physical and mental freedom/safety, physical environment (pollution, noise, traffic, climate), financial resources, opportunities to acquire new information and skills, opportunities for and participation in recreation and leisure, living conditions, health and the accessibility and quality of healthcare, and transport. The answers are on a 5-point scale (range scores 1–5). In each domain the maximum of 20 points is obtained. Individual result fields have a positive direction (the larger the number points, the higher the quality of life). The scoring of individual aspects has a positive dimension in that the higher the score, the better the quality of life [17]. Acceptable internal consistency was shown with Cronbach’s alpha coefficients being greater than 0.70 for all domains except for the social domain [20].

### 2.2. Organisation and Research Area

The study was conducted from December 2021 to February 2022 during the COVID-19 pandemic, in two forms. First, the survey was conducted using the online Google Forms on the Facebook social networking site ‘Niepochłonięci’ (Unlymphomised). Second, a paper questionnaire was distributed to patients of the hematology ward of a hospital in Krakow, Poland, after prior approval from the hospital director. Using Google Forms 51 questionnaires were collected, and 43 in hospital; 12 incomplete questionnaires were excluded from the statistical analyses. Finally, 82 complete survey questionnaires qualified for statistical analyses.

All respondents were informed about the research purpose and anonymity. Each respondent gave informed consent to participate in the study. The survey was conducted in accordance with the Declaration of Helsinki.

The sampling criteria were as follows: consent to participate in the survey, medical diagnosis of HL, undergoing systemic treatment, and mental state enabling understanding and completing the questionnaire independently. The exclusion criteria were the following: a lack of consent, undergoing diagnostics for lymphoma or completion of treatment (convalescents), and mental state preventing understanding and completing the questionnaire independently.

### 2.3. Statistical Analysis

The results obtained were analyzed using STATISTICA 10—an integrated statistical and analytical software package by StatSoft. Descriptive statistics (mean, median, standard deviation, minimum and maximum values) were used. The Kruskal–Wallis test, Mann–Whitney U test, Friedman test, and the Spearman rank-order correlation coefficient (Spearman’s rho) were used to evaluate the differences between the quantitative and qualitative variables (significance *p* < 0.05).

## 3. Results

The study involved 82 patients diagnosed with lymphoma, who met the inclusion criteria. The largest group (75.6%) were having their 4th to 7th cycles of chemotherapy, followed by 19.5% who were undergoing the 1st to 3rd cycles and 4.9% receiving the 8th to the 12th cycles. More than half of the patients (58%) stated they were additionally being treated for comorbidities. The most frequently mentioned diseases were degenerative changes of the spine (23%), arterial hypertension (18%), thyroid diseases (13%), diabetes (10%), and heart failure (7%).

The characteristics of the studied group are presented in Table 1.

**Figure 1:**
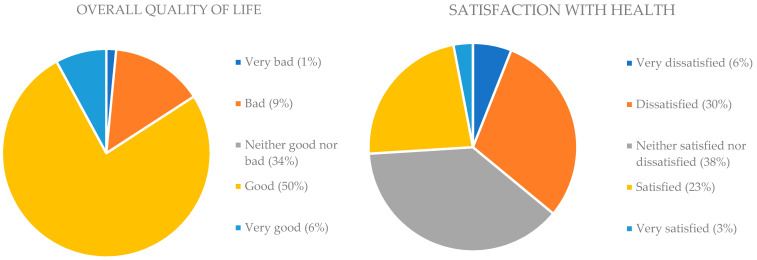
Surveyed patients’ subjective assessment of their overall quality of life and satisfaction with health.

The studied group included mostly women (77%), people aged up to 50 (69%), with secondary and higher education (85%), and living in a city (80%) with a family or partner (89%). More than half of the surveyed patients assessed their overall quality of life as good (50%) and very good (6%), while the expressed satisfaction with health most often ranged from neutral (38%—neither good nor bad) to dissatisfactory (30%) and very dissatisfactory (6%). Figure 1 shows the respondents’ general assessment of their quality of life and satisfaction with health.

**Figure 2:**
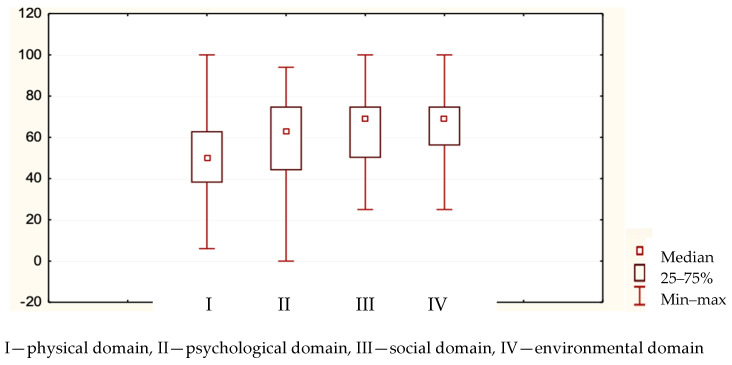
Graphical interpretation of the Mann–Whitney U test assessing the quality of life.

According to the key for calculating the results in the WHOQOL-BREF questionnaire, Table 2 and Figure 2 show the assessment results for individual domains.

The environmental domain was rated the highest, followed by the social and psychological domains, and the physical one scored the lowest. The analysis with the Mann–Whitney U test showed sex did not affect the assessment of the quality of life; the significance level was greater than 0.05.

In the study, we calculated the relationship between the assessment of the quality of life and demographic data. Table 3 shows the correlation between the assessment of the quality of life and age.

The Spearman’s rho obtained indicates there is no statistically significant correlation between the tested values, which means the assessment of the quality of life does not decrease or increase with the age of the respondents.

Further analysis shows, assuming the significance level of *p* < 0.05, that we have grounds to conclude that the assessment of the quality of life in the psychological domain (WHOQOL) significantly differs depending on the respondents’ place of residence. The median assessment of the quality of life in the psychological domain (WHOQOL) is the highest among people living in a city of fewer than 50,000 residents. No statistically significant difference was found in the remaining domains.

The environmental domain was rated the highest (64.30). Its components included: freedom/physical and mental security, physical environment, financial resources, opportunities to acquire new information and skills, opportunities for and participation in recreation and leisure, living conditions, health and accessibility, and quality of healthcare, and transport. Social relations were rated slightly lower (62.81).

Our study also showed no relationship between the assessment of the quality of life in individual domains and the education, place of residence, professional activity, and family status of the surveyed patients.

Almost all (96%) of the studied lymphoma patients treated systemically stated they received support during therapy. A detailed description of caregivers and the level of support received are presented in Table 4.

After applying the Friedman test and Kendall’s coefficient of concordance, we confirmed at a statistically significant level (*p* = 0.00000) the relationship between the level of support received and the providers of social support. Due to the insufficient size of the group, we did not examine the correlation between the assessment of the quality of life in individual domains and the support received. However, we obtained a statistically significant relationship (*p* = 0.005) in the Spearman’s rho between the support provided by the medical staff of the ward and the physical domain, which increased with the support provided. In the psychological domain, there was a statistically significant positive correlation with caregivers: family, friends, ward staff, and other people. A higher assessment of the psychological domain was associated with the sense of support received by respondents from the mentioned caregivers (Table 5). Similar dependencies were observed in the social and environmental domains.

Based on the obtained Spearman’s rho and the p index greater than 0.05, we showed that the stage of treatment, that is, the indicated cycle of chemotherapy, did not affect the assessment of the quality of life.

The studied group of patients with lymphoma was treated systemically. Therefore, the research subject was the assessment of the side effects of treatment that might occur during hospitalization (Table 6) and when at home between successive cycles of chemotherapy (Table 7).

The most frequently indicated and also the most troublesome side effects during hospitalization were hair loss (44% of respondents), weakness (32%), and nausea (13%). All patients complained of weakness during hospitalization, and only three (4%) did not experience this at home (Table 7).

The study shows that when staying at home, patients most often complained of weakness (sometimes, often, and very often—96% of respondents) and hair loss (83%), followed by a lack of appetite (51%), insomnia (43%), and constipation (38%). At the same time, the study showed hair loss did not have a statistically significant effect on any of the domains depicting the respondents’ assessment of the quality of life.

The analysis of the Kruskal–Wallis rank test revealed the physical domain was statistically significantly affected by the following side effects of treatment: weakness (*p* = 0.0113 during hospitalization and *p* = 0.0010 at home), diarrhea (*p* = 0.0254 during hospitalization and *p* = 0.0030 at home), constipation (*p* = 0.0046 during hospitalization and *p* = 0.0242 at home), and nausea (*p* = 0.0482 during hospitalization). The psychological domain was influenced by such symptoms as diarrhea occurring at home (*p* = 0.0256), constipation in hospital (*p* = 0.0199), and nausea during hospitalization (*p* = 0.0220). In the environmental domain, nausea (*p* = 0.0011 during hospitalization and *p* = 0.0184 at home) had a statistically significant effect.

The Spearman’s rho obtained and the p index greater than 0.05 showed that the stage of treatment, that is, the cycle of chemotherapy indicated by the examined patients, did not affect the assessment of the quality of life.

The study of the relationship between the assessment of the quality of life and the occurrence of comorbidities showed a statistically significant correlation with the physical domain (Z = −2.63071, *p* = 0.008) and the environmental domain (Z = −2.1175, *p* = 0.034). This means that people suffering from additional diseases assessed the quality of life in these domains as lower than those with no diseases other than lymphoma.

## 4. Discussion

Cancer occupies one of the leading places on the list of lifestyle diseases. Modern achievements of medicine in the field of oncology offer a wide range of activities for diagnostics and anticancer treatment. The multidimensional approach to the oncological patient puts deep emphasis on the patient’s feelings, the comfort of life, and the bio-psycho-social aspect of their functioning, in general the quality of life. The subjective assessment of the quality of life made by patients allows medics to see the impact of treatment and oncological care on the patient and their relatives.

HRQL has been explored by many researchers, including those specializing in psycho-oncology, in their scientific works. For example, De Walden-Gałuszko emphasizes that HRQL includes the patient’s overall assessment of the disease and treatment in physical, mental, and social areas [21].

The research subject aim was to examine the quality of life of patients with HL undergoing anticancer therapy. The studied group consisted of 82 patients during various cycles of systemic treatment. They assessed their overall quality of life as very good and good (56%), while 36% were not satisfied with their general health. Our study shows that sex, age, and level of education did not have a statistically significant effect on the overall assessment of the quality of life. Despite the much larger number of women in our study, there was no statistical dependence. The larger number of women in relation to men is probably because women more often take care of their health and are more willing to undergo diagnostic tests. Interesting results were revealed in the perception of the psychological domain among respondents living in small towns. More advancement in the care for the sick can be seen in cities, especially smaller ones. In many villages in Poland, cancer is still treated as a stigma, which is why patients do not talk about their disease and, consequently, do not receive psychological help. Similar results were obtained by Zhang et al., who noticed older people from rural areas had worse HRQL conditions compared to people living in urban areas [22]. Ślusarska et al. confirmed a lower level of acceptance of the disease in rural residents compared to urban ones and showed that men assessed the quality of life in different ways, while women usually declared a medium degree of perceived quality of life [23].

When analyzing the values of the particular domains of quality-of-life assessment, it can be seen the physical domain was rated the lowest. Side effects related to the treatment and the disease had a direct impact on the values—both during hospitalization and at home, weakness was the dominant side effect in the study. Many authors indicate this complication is common and burdensome [24,25,26]. The side effects of chemotherapy can be alleviated by psychotherapy and physical exercise. In the case of our patients, especially during the pandemic, these activities were not carried out comprehensively. Lewandowska’s research on the quality of life during chemotherapy confirmed the impact of treatment and related side effects on the reduction in the quality of life of cancer patients [27]. Similarly, Rosińczuk et al. observed a low quality of life in patients undergoing chemotherapy due to the occurrence of side effects [28]. Milaniak et al. also found that chemotherapy decreased the quality of life compared to the state before treatment in about 30% of patients, while 79% experienced significant treatment-related fatigue [29]. The results of our own research indicate the need to undertake or periodically review activities related to the scope of treatment supporting the patient during therapy. In Poland, it was only last year that a statutory obligation to appoint the position of “oncological care coordinator” was introduced. His responsibilities include not only managing the treatment path, but also monitoring patient problems and referring the patient to appropriate specialists.

The respondents constituted a relatively young, studied group and so 43% of them did not declare any comorbidities. In the remaining group, our study makes it possible to conclude that the assessment of the quality of life differs significantly in the physical and environmental domains (WHOQOL), depending on the presence of comorbidities. We claim the results of the assessment of the quality of life in the physical and environmental domains (WHOQOL) are lower among people declaring the presence of comorbidities, compared to those without intercurrent diseases.

The environmental domain was rated the highest and social relations were rated slightly lower (62.81). These aspects are directly related to social support, needed by an ill person to fight for health, and are invaluable. Our study showed that a family was the basic source of support, especially emotional, but also informative and financial. Similar results were obtained by Zielińska-Więczkowska et al., who showed that multidimensional support is important in cancer, and that it was the closest family that provided fundamental support during treatment [30]. The obtained results indicate the absolute necessity to include caregivers in the therapeutic team. This will involve not only improving the patient’s quality of life and a better course of therapy, but also social support for the caregiver.

## 5. Limitations

The study was cross-sectional and concerned only patients. It was conducted during the pandemic, which resulted in the decision to select fewer research tools and forced the need to accept a smaller size of the study group.

Analyzing the problems and needs of patients suffering from T-cell and B-cell lymphomas and the constantly emerging new discoveries in the area of quality of life and social support in oncology, an important recommendation seems to be the proposal to conduct longitudinal research on the oncological patient—caregiver dyad (the closest person indicated by the patient) [31]. Such studies could be planned before the start of treatment, at certain stages of treatment, and immediately, at one year, and at five years after the end of treatment.

## 6. Conclusions

Cancer—a disease that in the public perception still causes anxiety and fear, requires systematic research on the quality of life, coping with the healing process and multidimensional social support. The results obtained become the basis for evidence-based nursing care planning.

In this study, the patients rated the physical domain the lowest, confirming the occurrence of side effects associated with systemic treatment, both during hospitalization and at home.

The quality of life in our study group did not correlate at a statistically significant level with socio-demographic variables, i.e., age, sex, level of education and the occurrence of comorbidities.

Studies have shown a statistically significant positive correlation between the assessment of the quality of life of our patients in all WHOQOL domains and the degree of support received from informal and formal caregivers. This area may be the subject of future research.

## Figures and Tables

**Table 1:** Sociodemographic characteristics of the studied group.

Variables		N Respondents	% Respondents
Sex	Female	63	76.82
	Male	19	23.18
Age (years)	20–30	20	24.40
	31–40	23	28.05
	41–50	14	17.07
	51–60	7	8.54
	61–70	14	17.07
	Over 70	4	4.87
Education	Preliminary	4	4.87
	Vocational	8	9.75
	Secondary	25	30.49
	Higher	45	54.89
Place of residence	City of more than 50,000 residents	44	53.66
	City of fewer than 50,000 residents	22	26.83
	Village	16	19.51
Work activity	Professional work	23	28.05
	Sick leave	23	28.05
	Retirement/pension	23	28.05
	Other	13	15.85
Family status	Lives with a family	60	73.17
	Lives with a partner	13	15.85
	Lives alone	9	10.98

**Table 2:** Quality of life in particular domains as assessed by the surveyed patients.

WHOQOL Domains	Average Rank	Sum of Ranks	Mean [0–100]	Standard Deviation
Physical	1.80	147.5	51.32	19.17
Psychological	2.58	211.5	60.38	20.18
Social	2.73	224.0	62.82	17.86
Environmental	2.89	237.0	64.30	16.05

Friedman’s test, ANOVA and Kendall’s coefficient of concordance. Chi-squared, ANOVA (N = 82, df 3) = 37.71713, *p* = 0.00000. Coefficient of concordance = 0.15332 r average rank = 0.14287.

**Table 3:** Spearman’s rho for quality of life and age assessment.

Age and WHOQOL Domains	N Significant	Spearman’s Rho	T (n − 2)	*p*
physical	82	−0.03	−0.25	0.80
psychological	82	0.54	0.49	0.68
social	82	0.061	0.54	0.59
environmental	82	0.068	0.61	0.54

**Table 4:** Descriptive statistics of the scale of support received by respondents.

Caregivers	Mean	Median	Min	Max	Lower Quartile	Higher Quartile	Standard Deviation
Family	6.16	7	1	7	6	7	1.48
Friends	5.32	6	1	7	4	7	1.77
Institutions	1.45	1	1	7	1	1	1.12
Hematology staff	4.55	5	1	7	4	6	1.83
Other people	2.63	2	1	7	1	4	1.84

**Table 5:** Spearman’s rho and caregivers.

Caregivers Psychological Domain	N Significant	Spearman’s Rho	T (n − 2)	*p*
Family	82	0.38	3.63	0.00
Friends	82	0.35	3.36	0.00
Ward staff	82	0.41	4.03	0.00
Institutions	82	0.01	−0.08	0.93
Other	82	0.33	3.12	0.00

**Table 6:** Symptoms occurring during hospitalization in connection with treatment and disease.

Side Effects during Hospitalization	Never		Sometimes		Often		Very Often	
	[n]	[%]	[n]	[%]	[n]	[%]	[n]	[%]
Weakness	0	0	24	29	32	39	26	32
Diarrhea	51	62	22	27	8	10	1	1
Constipation	23	28	36	44	14	17	9	11
Nausea	24	29	36	44	11	13	11	13
Vomiting	47	57	26	32	5	6	4	5
No appetite	24	29	41	50	13	16	4	5
Skin lesions	39	48	29	35	8	10	6	7
Hair loss	8	10	13	16	25	30	36	44

**Table 7:** Symptoms occurring at home in connection with treatment and disease.

Side Effects at Home	Never		Sometimes		Often		Very Often	
	[n]	[%]	[n]	[%]	[n]	[%]	[n]	[%]
Weakness	3	4	25	30	27	33	27	33
Diarrhea	51	62	22	27	8	10	1	1
Constipation	29	35	31	38	15	18	7	9
Nausea	28	34	29	35	15	18	10	12
Vomiting	50	61	21	26	7	9	4	5
No appetite	29	35	42	51	9	11	2	2
Weight loss	39	48	27	33	13	16	3	4
Hair loss	14	17	15	18	27	33	26	32
Memory problems	23	28	24	29	23	28	12	15
Insomnia	20	24	35	43	17	21	10	12
Limited mobility	23	28	30	37	19	23	10	12

## Data Availability

The data presented in this study are available on request from the corresponding author.

## Public Involvement Statement

There was no public involvement in any aspect of this research.

## Guidelines and Standards Statement

This manuscript was drafted against the STROBE for cross-sectional studies.
